# The relation of orthorexia with lifestyle habits: Arabic versions of the Eating Habits Questionnaire and the Dusseldorf Orthorexia Scale

**DOI:** 10.1186/s40337-021-00455-z

**Published:** 2021-08-14

**Authors:** Souheil Hallit, Juan Ramón Barrada, Pascale Salameh, Hala Sacre, María Roncero, Sahar Obeid

**Affiliations:** 1 Faculty of Medicine and Medical Sciences, Holy Spirit University of Kaslik (USEK), Jounieh, Lebanon; 2 INSPECT-LB, Institut National de Santé Publique, Épidémiologie Clinique et Toxicologie, Beirut, Lebanon; 3 Facultad de Ciencias Sociales y Humanas, Universidad de Zaragoza, Teruel, Spain; 4 Faculty of Pharmacy, Lebanese University, Hadat, Lebanon; 5 University of Nicosia Medical School, Nicosia, Cyprus; 6 Facultad de Psicología, Universitat de València, Valencia, Spain; 7 Research and Psychology Departments, Psychiatric Hospital of the Cross, Jal Eddib, Lebanon; 8 Faculty of Arts and Sciences, Holy Spirit University of Kaslik (USEK), Jounieh, Lebanon

**Keywords:** Orthorexia nervosa, Healthy behaviors, Alcohol, Smoking, Physical activity

## Abstract

**Background:**

Some of the commonly used tools to assess orthorexia nervosa (OrNe) do not allow a meaningful interpretation of the scores or yield mixed results about the dimensions needed to represent orthorexia. Since no advancement in the theoretical knowledge can be made without a thorough examination of the measurement aspects, this study aimed to evaluate the correlation between orthorexia nervosa (OrNe) and lifestyle habits, notably alcohol drinking, cigarette and waterpipe smoking, and physical exercise, and to validate and assess the psychometric properties of the Arabic versions of the Eating Habits Questionnaire (EHQ) and Düsseldorf Orthorexia Scale (DOS).

**Methods:**

A total of 456 adult participants completed a self-administered questionnaire. Exploratory structural equation models were used to test the internal structure of the instruments. Shorter and more explicit versions were proposed for instruments. Pearson and partial correlations were computed between orthorexia scores and healthy behaviors scores.

**Results:**

Regarding the internal structure of both EHQ and DOS, evidence favored the bi-dimensional construct of orthorexia. Both tools presented two theoretically clearly interpretable factors (OrNe and Healthy Orthorexia—HeOr—). The two questionnaires presented a high convergent validity, as dimensions with the same interpretation were correlated around 0.80. While OrNe was positively correlated with the use of unhealthy substances (higher alcohol use disorder, cigarette, and waterpipe dependence), HeOr was negatively associated with these behaviors.

**Conclusion:**

Our results emphasize the idea that further attention should be paid to the multidimensional structure of orthorexia, as OrNe and HeOr present an opposite pattern of associations with healthy behaviors. An OrNe etiopathogenesis common to eating disorders can explain these differences.

## Plain English summary

The term “orthorexia nervosa” (OrNe) denotes a possible pathological fixation on a health-conscious diet. Some of the commonly used tools to assess orthorexia nervosa (OrNe) do not allow a meaningful interpretation of the scores or yield mixed results about the dimensions needed to represent orthorexia. In this study, some items were simultaneously tapping both dimensions of orthorexic tendencies, and shorter versions of the DOS and EHQ scales were proposed to offer instruments with a more explicit structure. The final versions still presented high reliabilities. While HeOr was negatively associated with the consumption of unhealthy substances (higher alcohol use disorder, cigarette, and waterpipe dependence), OrNe was positively correlated with these behaviors. After controlling for OrNe or HeOr (partial correlations), the associations were even higher; the pattern of associations was more explicit, showing the importance of considering the multidimensional structure of orthorexia.

## Background

The term "orthorexia nervosa" (OrNe) denotes a possible pathological fixation on a health-conscious diet. The Greek origin of "orthorexia" ("orthós" = right or correct and "orexsis" = hunger or appetite) outlines the obsession for healthy and right food [1, 2]. At present, although the classification of OrNe in the Diagnostic and Statistical Manual of Mental Disorders (DSM-5) is still under debate [3], several proposals have been made to make it a distinct subtype of avoidant/restrictive food intake disorder [4], sharing characteristics with anorexia nervosa [5] or overlapping with obsessive–compulsive disorder [6]. Despite increased research on this subject in recent years, the literature on OrNe is still mostly descriptive, with often inconsistent results in terms of risk and protective factors [7].

Eating behavior is a complex process, including individual perception, nutritional status, social, demographic, and cultural conditions, among others [8, 9]. People with OrNe tendencies and behaviors are supposed to have a high commitment to perfect physical health [10]. Healthy eating would be a part of this general goal, but other lifestyle features should be considered. Thus, higher levels of physical activity were seen in participants with higher OrNe scores [11–13]. From this perspective, OrNe should be positively related to healthier behaviors.

From another perspective, a negative relation between OrNe and a healthy lifestyle could be anticipated, considering the similarity of OrNe with some eating disorders. Disordered eating has been linked to substance use disorders [14, 15]—although the evidence is mixed for anorexia nervosa [14, 15]–, particularly alcohol [16] and cigarettes [17]. Indeed, lower OrNe tendencies and behaviors were seen in people who never drank alcohol compared to alcohol users [18]. Moreover, women with disordered eating use smoking as a method to control weight [19], as nicotine suppresses appetite and increases resting metabolic rate [20]. OrNe is more closely related to weight control motives for food choice than to health content motives [21], a result that casts doubts about the real connection between OrNe and healthy lifestyle choices.

To date, advancement in knowledge about OrNe has been, to a certain extent, hindered by the way orthorexia was assessed. Multiple scales have been used to screen for orthorexic tendencies and behaviors among people: orthorexia self-test (BOT), the ORTO-15 questionnaire, Eating Habits Questionnaire (EHQ), Düsseldorf Orthorexia Scale (DOS), Teruel Orthorexia Scale (TOS), Barcelona Orthorexia Scale (BOS), and Orthorexia Nervosa Inventory (ONI) [22]. The most commonly used scale, the ORTO-15 tool [2, 3], was created and validated in Italy [23]. Nowadays, there is a growing consensus that the ORTO-15 is not a valid questionnaire [24, 25], and thus, that results from studies using this tool should be considered with caution [26, 27].

Other tools with better psychometric properties but with still relevant limitations have been developed to assess OrNe. The EHQ [28], created by Gleaves et al. in a population from the USA (two studies, *n*s = 174 and 213), assesses cognitions, behaviors, and feelings related to an extreme focus on healthy eating. The DOS [29] was created by Barthels et al. in a German population (*n* = 1340). Both the EHQ and the DOS present some contradictory results in terms of their internal structure.

The EHQ was initially designed and validated to measure three factors [28]: Problems Associated With Healthy Eating, Knowledge of Healthy Eating, and Feeling Positively About Healthy Eating. Some studies could not replicate this structure [27, 30–32]. On a sample of 459 participants from the USA, Oberle et al. [30] recovered three slightly different factors (Behaviors, Problems, and Feelings), five items having relevant cross-loadings (loadings over |.30|) in a secondary dimension. Godefroy et al. [32] chose a solution with four factors (Rigid Eating Behavior, Positive Feeling of Control, Problem of Attention Control, and Social Relationships) on a sample of 1887 French participants and removed five items from the original EHQ. Halim et al. [31] conducted a study on 286 Australian women and retained a four factors solution: Healthy Eating Cognitions, Dietary Restriction, Diet Superiority, and Social Impairment. In those results, eight items presented relevant cross-loadings, in some cases, almost the same size as the primary loadings. Even for solutions with the same number of factors, the distribution of items per factor was not equivalent, as was their theoretical interpretation. Meule et al. [33] have replicated the original structure on a German sample of 511 participants, although no other models were tested.

The DOS has been validated on university students in English [34] (sample from the USA, *n* = 384), Spanish [35] (sample from Spain, *n* = 492), and Chinese [36] (sample from China, n = 1075). Data did not support the presence of a single dimension for the German, English, and Chinese versions of the scale. In the Spanish version, a unidimensional model offered an excellent fit. For the Chinese version, a three-dimensional solution was considered, with factors labeled Obsession in Healthy Food, Adherence to Nutrition Rules, and Emotional Symptoms. Meule et al. [33] obtained a good fit for the unidimensional solution. In that study, total scores of the EHQ and the DOS showed a high correlation (*r* = 0.79).

As the different validations studies vary in so many aspects, it is not possible to establish clearly why those differences in the internal structure of the questionnaires are found. These differences could be due to the translation process, cultural differences, differences in the composition of the samples (ages, genders, education levels…), among other reasons. Importantly, although there is some evidence about the multidimensionality of these questionnaires, it is still common to compute a single total score, which will mask the possible different associations of these various latent factors. These inconsistencies also point to the need for further efforts to understand what the EHQ and the DOS are measuring.

The vast majority of the studies about OrNe have been conducted in Western countries rather than non-Western ones. However, according to the values and attitudes connecting individuals to their social group, cultures can also be divided into collectivistic and individualistic [37]. Collectivistic cultures emphasize the individual’s behavior for the whole group, focusing on cooperative tasks, whereas individualistic societies are characterized by an emphasis on what makes the individual distinct, focusing on competitive tasks [38]. Some of the characteristics of the collectivistic culture such as high levels of parental overprotection, social pressure resulting from the standards of female beauty imposed by industrial society or Western culture [39], change in cultural environment (i.e., exposure to modernity and experimentation with individualistic models) might lead Eastern countries (like Lebanon) to develop OrNe in reaction to socio-cultural disconnection and transition.

The aims of this study were twofold. First, to provide further evidence about the internal structure of the Arabic versions of the EHQ and DOS. This goal was deemed relevant for three reasons: (a) the internal structure of these instruments is still unclear, (b) the extent to which the different OrNe questionnaires are addressing the same construct has not been fully established, and (c) research about OrNe in Eastern cultures is scarce. Second, to evaluate the correlation between OrNe and lifestyle habits, notably alcohol drinking, cigarette and waterpipe smoking, and physical exercise, and test the association between orthorexic scores and some basic sociodemographic information, like age and gender. The associations of OrNe and gender are unclear as much of the previous research has been based on the ORTO-15 [40].

As previously described, some of the commonly used instruments for the assessment of OrNe do not allow a meaningful interpretation of the scores or yield mixed results regarding how many dimensions are needed to represent orthorexia. Given this situation, it is not possible to advance in the theoretical knowledge of OrNe and its relationship with lifestyle habits without progressing in the psychometric/instrumental research about orthorexia. The present manuscript addresses both elements simultaneously, as, currently, no theoretical progress can be made without a thorough examination of the measurement aspects.

## Methods

### Participants

The initial sample was of 519 (82.38%) out of 630 participants approached. As inclusion criteria, participants had to: (a) be aged 18 years and above, and (b) present less than four missing values in the EHQ responses and less than two missing values in the DOS responses. Those somehow arbitrary cut-points were defined so that more than 80% of the responses were provided. In the final sample, the mean number of missing values for the EHQ responses was 0.14 items and for the DOS responses 0.09 items. After applying these criteria, the final sample consisted of 456 participants, of whom 250 were women (56.2%), 194 men (43.6%), and one unreported gender (0.2%). Mean age was 37.02 years (SD = 13.85, range [18, 75]). The same sample and methodology were used in a previous paper [41]. All methods were performed in accordance with the relevant guidelines and regulations.

### Translation procedure of the questionnaire

A forward and backward translation was performed for all the scales by two translators, one for the translation from English into Arabic, and the other for the back-translation. During the forward translation phase, the principal emphasis was to reach equality between the English and Arabic versions while using a comprehensible vocabulary. The Arabic form was revised by an expert committee composed of the original translator, one psychiatrist, and two psychologists [42, 43]. The same process was used in the back-translation from Arabic into English. Discrepancies between the original and translated English versions were resolved by consensus [44, 45]. A pilot study was conducted on 20 persons to ensure that the questions are well understood; no significant changes were made to the Arabic version subsequently, thus, the results were included in the final dataset.

### Procedure

The sample was recruited from seven community pharmacies randomly chosen from a list provided by the Lebanese Order of Pharmacists, the official pharmacists’ association in Lebanon. A simple randomization process was followed. Each person entering a pharmacy was encouraged to participate in the study. Well-trained interviewers explained the study objectives for each participant. At the end of the process, the completed questionnaires were collected back by the interviewers and sent for data entry. The anonymity of participants was guaranteed by putting filled out questionnaires into closed boxes.

### Questionnaires and variables

The self-administered battery of questionnaires was anonymous and available in Arabic, the mother tongue in Lebanon.

#### Sociodemographic data

Participants provided information about their age, gender and physical activity. The Physical Activity Index was calculated by multiplying the reported intensity (from 1 = *light (fishing, walking)* to 5 = *sustained heavy breathing and perspiration*), duration (from 1 = *under 10 min* to 4 = *over 30 min*), and frequency of daily activity (from 1 = *less than once a month* to 5 = *daily or almost daily*) [46].

#### Eating Habits Questionnaire (EHQ)

The EHQ is a 21-item self-reported questionnaire to measure orthorexic eating behavior. Answers are scored on a four-point scale from 1 = *false, not at all true* to 4 = *very true *[28]. Cronbach's alpha in the current sample was equal to 0.940.

#### Düsseldorf Orthorexia Scale (DOS)

The DOS is a 10-item self-reported questionnaire to measure orthorexic eating behavior. Answers are scored on a four-point Likert-scale from 1 = *this does not apply to me* to 4 = *this applies to me *[29]. Cronbach's alpha in the current sample was equal to 0.896.

#### Alcohol Use Disorders Identification Test (AUDIT)

The self-reported version of this 10-item tool was used in this study to assess alcohol use, drinking patterns, and alcohol-related issues [47]. Higher scores would indicate more alcohol use disorder. Cronbach's alpha in the current sample was equal to 0.96.

#### Fagerström Test for Nicotine Dependence (FTND)

The FTND, in its Arabic version [48], consists of six items, scored as 0/1 for the no/yes questions and from 0 to 3 for multiple-choice items [49]. Higher scores would indicate higher cigarette dependence. Cronbach's alpha in the current sample was equal to 0.82.

#### Lebanon Waterpipe Dependence Scale (LWDS)

The waterpipe involves “the passage of charcoal-heated air through a perforated aluminum foil and across the flavored tobacco to become smoke that bubbles through the water before inhalation by the smoker” [50]. LWDS test was used to assess waterpipe dependence [51]. It includes 11 items scored from 0 to 3. Higher scores would indicate higher waterpipe dependence. Cronbach's alpha in the current sample was equal to 0.88.

### Statistical analysis

The internal structure of the EHQ and DOS scores were analyzed separately, with exploratory structural equation models (ESEM) [52, 53]. This technique was preferred, given the uncertainty about the factor structure of those questionnaires. Parallel analysis [54] and visual inspection of the scree-plot were used, in addition to the results from previous studies, theoretical interpretability of the solutions, factor simplicity, and loading sizes to determine the number of dimensions to be retained, which could not be anticipated before data analysis.

Models were analyzed using robust weighted least squares (WLSMV estimator in M*Plus*). According to conventional cut-offs [55], CFI and TLI with values greater than 0.95 and RMSEA less than 0.06 were indicative of a satisfactory fit. It should be noted, first, that those cut-offs were developed for confirmatory factor analysis with continuous responses and, second, that these cut-off values should be treated as rough guidelines and not interpreted as "golden rules" [56], so those values should be considered with caution. The authors are not aware that specific cut-offs have been proposed for ESEM with categorical variables. Localized areas of strain were assessed with modification indices (MI). The standardized solution (STDYX solution in M*Plus*) was interpreted for all the factor models, and the default rotation in M*Plus*, Geomin, was applied.

Items for which (a) loadings in all the factors were below |0.50|, or (b) more than a single loading was above |0.30| [57], were retained from the EHQ and the DOS to develop a final shortened orthorexia questionnaires with more explicit factor structures. Reliabilities were estimated with Cronbach’s alpha.

After defining the final shortened version of the EHQ and DOS, the internal structure of those questionnaires was analyzed simultaneously. By doing so, the correlation between the latent factors assessed by both questionnaires and the convergent validity could be computed. In this ESEM model, the EHQ defined the first set of factors, and the DOS defined the second set. The solution that was considered as preferable in the previous analysis was used for the EHQ and DOS structures.

Pearson correlations were computed between the different dimensions of the EHQ and the DOS scores and the other measures (lifestyle habits, gender, and age). When those instruments turned out to be multidimensional, partial correlations were computed between the various factors and the other variables while controlling for the rest of the orthorexia factors. Partial correlations were preferred over other analytical alternatives as regression models—perhaps more commonly reported—for several reasons. First, Pearson and partial correlation are more easily comparable, as both can range from − 1 to + 1, while this is not the case for regression coefficients (even for standardized coefficients). Second, partial correlations and regression coefficients lead to the same inferential results, as both share identical *p*-values. Third, there was no justification for the inclusion of additional covariates in the analysis.

The analysis was performed with M*Plus* 7.4 [58] and R 3.6.1 [59], with packages *psych* version 1.8.12 [60] and *MplusAutomation* version 0.7 [61].

## Results

### Psychometric properties of the orthorexia questionnaires

#### EHQ

**Fig. 1:**
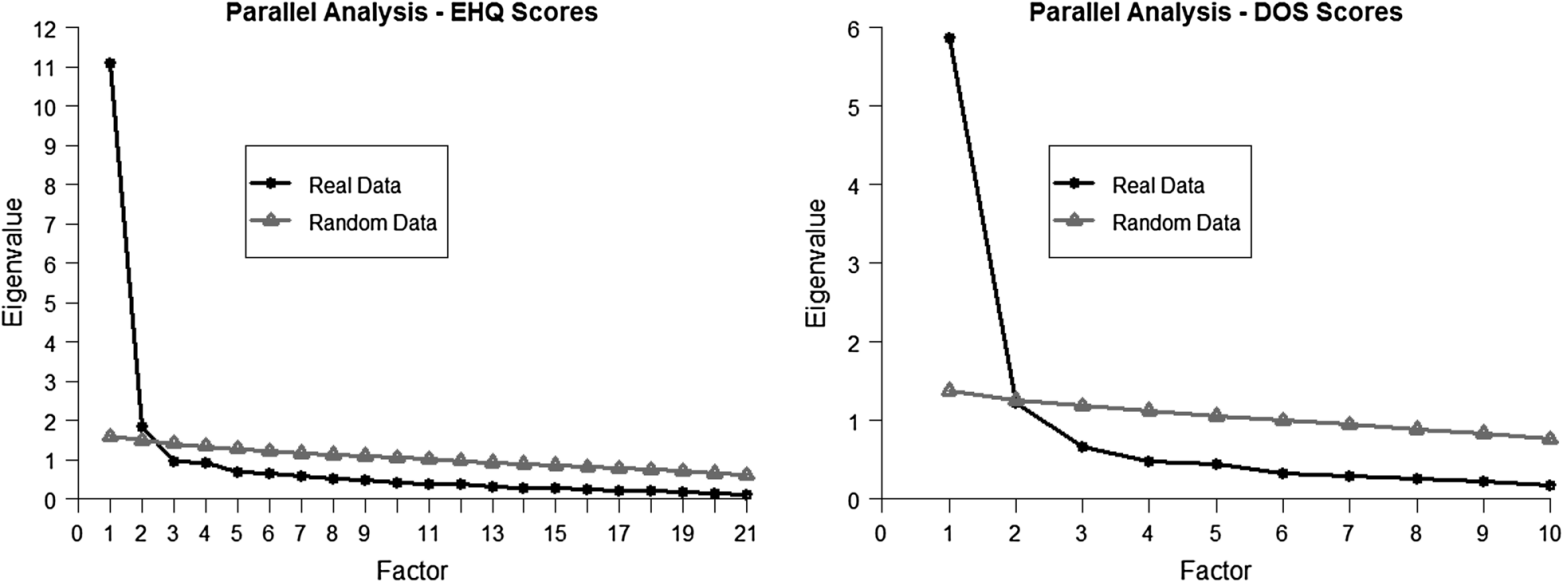
Parallel analysis of the Eating Habits Questionnaire (EHQ) and Dusseldorf Orthorexia Scale (DOS) scores

For EHQ scores, both the scree-plot and the parallel analysis (shown in Fig. 1) suggested the convenience of retaining two factors. As the majority of previous studies retained three factors, solutions were compared to the two number of factors. TLI and RMSEA values (shown in Table 1) indicated misfit for both options, while CFI value did for the two-factor solution (two factors/three factors: CFI = 0.944/0.962, TLI = 0.930/0.946, RMSEA = 0.079/0.070). After the inspection of item loadings, the two-factor solution was selected, as in the case of three factors no item presented relevant loading in the last factor, so this third factor could not be interpreted.

**Table 1:** Goodness of fit indices for the different models

Model	*χ*^2 †^	*df*	CFI	TLI	RMSEA
M1. EHQ Two Factors	647.8	169	.944	.930	.079
M2. EHQ Three Factors	476.8	150	.962	.946	.070
M3. EHQ Two Factors Short Version	307.5	76	.959	.943	.082
M4. EHQ Two Factors Short Version CU E15–E19	260.7	75	.967	.954	.074
M5. DOS One Factor	507.6	35	.894	.863	.173
M6. DOS Two Factors	158.3	26	.970	.948	.106
M7. DOS Two Factors CU D04 & D07	117.8	25	.979	.962	.091
M8. DOS Two Factors Short Version CU D04–D07	79.3	18	.984	.967	.087
M9. Final EHQ Model & Final DOS Model	587.8	224	.959	.949	.060

df, degrees of freedom; CFI, comparative fit index; TLI, Tucker-Lewis index; RMSEA, root mean square error of approximation; CU, correlated uniquenesses; EHQ, Eating Habits Questionnaire; DOS, Dusseldorf Orthorexia Scale

^†^All p-values for the *χ*^2^ test were < 0.001

From the initial two factor solution (shown in Table 2), six items were removed due to the presence of two loadings over |0.30|. Inspection of the modification indices showed that one of them stood out above the others (MI = 48.3), indicating the convenience of allowing items uniqueness from Item 15 ("Eating the way I do gives me a sense of satisfaction") and Item 19 ("I feel great when I eat healthily"). The overlap between “satisfaction” and “feel great” was considered enough to make this new parameter theoretically interpretable. After the inclusion of this correlated uniqueness, a satisfactory model fit was achieved, except for RMSEA (CFI = 0.967, TLI = 0.954, RMSEA = 0.074).

**Table 2:** Item Loadings of the Initial and Short Version of the Eating Habits Questionnaire (EHQ)

	Initial version		Short version	
	HeOr	OrNe	HeOr	OrNe
E01. I am more informed than others about healthy eating	**0.79**	− 0.05	**0.80**	− 0.03
E02. I turn down social offers that involve eating unhealthy food	**0.62**	0.11	**0.64**	0.12
E03. The way my food is prepared is important in my diet	**0.82**	− 0.12	**0.81**	− 0.11
*E04. I follow a diet with many rules	**0.51**	**0.31**	–	–
*E05. My eating habits are superior to others	**0.31**	**0.52**	–	–
E06. I am distracted by thoughts of eating healthily	0.00	**0.73**	0.01	**0.72**
E07. I only eat what my diet allows	0.18	**0.63**	0.18	**0.63**
E08. My healthy eating is a significant source of stress in my relationships	− 0.19	**0.97**	− 0.16	**0.96**
*E09. I have made efforts to eat more healthily over time	**0.50**	**0.33**	–	–
E10. My diet affects the type of employment I would take	− 0.22	**0.94**	− 0.20	**0.93**
E11. My diet is better than other people’s diets	**0.78**	0.06	**0.79**	0.07
E12. I feel in control when I eat healthily	**0.80**	0.00	**0.80**	0.01
E13. In the past year, friends or family members have told me that I’m overly concerned with eating healthily	0.19	**0.65**	0.22	**0.64**
*E14. I have difficulty finding restaurants that serve the foods I eat	**0.39**	**0.38**	–	–
E15. Eating the way I do gives me a sense of satisfaction	**0.72**	0.02	**0.64**	0.05
*E16. Few foods are healthy for me to eat	**0.37**	**0.34**	–	–
E17. I go out less since I began eating healthily	0.16	**0.63**	0.18	**0.62**
E18. I spend more than three hours a day thinking about healthy food	0.06	**0.75**	0.08	**0.74**
E19. I feel great when I eat healthily	**0.58**	0.19	**0.46**	0.24
E20. I follow a health-food diet rigidly	0.17	**0.69**	0.16	**0.68**
*E21. I prepare food in the most healthful way	**0.42**	**0.42**	–	–

HeOr, Healthy Orthorexia; OrNe, Orthorexia Nervosa; Bold values indicate loadings, in absolute value, over0.30. – indicates items not included in the short version. Item wording with as asterisk correspond to items not included in the short version

In this final solution, the two factors were interpreted as Healthy Orthorexia (HeOr; seven items; e.g., “The way my food is prepared is important in my diet”) and OrNe (eight items; e.g., “My healthy eating is a significant source of stress in my relationships”). Both factors correlated to 0.60. Cronbach's alpha was 0.86 and 0.89 for EHQ HeOr and EHQ OrNe, respectively.

#### DOS

For DOS scores, both the scree-plot and the parallel analysis were unclear, indicating one or two factors as appropriate decisions. Both options were compared. While model fit was poor for the unidimensional solution (CFI = 0.894, TLI = 0.863, RMSEA = 0.173), it was greatly improved in the two-factor solution (CFI = 0.970, TLI = 0.948, RMSEA = 0.106), although with relevant misfit still present. In this solution, a MI stood out (MI = 41.8), suggesting the convenience of allowing the correlation between Item 4 and Item 7 uniqueness. Both items tapped the negative social impact of a rigid diet. After the inclusion of this new parameter, a satisfactory model fit was achieved, except for RMSEA (CFI = 0.979, TLI = 0.962, RMSEA = 0.091).

From this two-factor solution, a single item was removed (“I like that I pay more attention to healthy nutrition than other people”), as it presented loadings over |0.30| in both factors (Table 3). Model fit for the shortened version was adequate, again except for RMSEA (CFI = 0.984, TLI = 0.967, RMSEA = 0.087).

**Table 3:** Item loadings of the initial and short version of the Dusseldorf Orthorexia Scale (DOS)

	Initial version		Short version	
	HeOr	OrNe	HeOr	OrNe
D01. Eating healthy food is more important to me than indulgence/enjoying the food	**0.81**	0.01	**0.84**	− 0.01
D02. I have certain nutrition rules that I adhere to	**0.84**	− 0.02	**0.80**	0.00
D03. I can only enjoy eating foods considered healthy	**0.66**	0.17	**0.67**	0.17
D04. I try to avoid getting invited over to friends for dinner if I know that they do not pay attention to healthy nutrition	0.00	**0.67**	− 0.01	**0.68**
*D05. I like that I pay more attention to healthy nutrition than other people	**0.52**	**0.36**	–	–
D06. If I eat something I consider unhealthy, I feel really bad	0.16	**0.71**	0.11	**0.73**
D07. I have the feeling of being excluded by my friends and colleagues due to my strict nutrition rules	− 0.29	**0.95**	− 0.27	**0.93**
D08. My thoughts constantly revolve around healthy nutrition and I organize my day around it	− 0.01	**0.86**	0.00	**0.86**
D09. I find it difficult to go against my personal dietary rules	0.00	**0.81**	0.02	**0.80**
D10. I feel upset after eating unhealthy foods	0.06	**0.71**	0.08	**0.71**

HeOr, Healthy Orthorexia; OrNe, Orthorexia Nervosa; Bold values indicate loadings, in absolute value, over0.30. – indicates items not included in the short version. Item wording with as asterisk correspond to items not included in the short version

In this final solution, the two factors were again interpreted as HeOr (three items; e.g., “Eating healthy food is more important to me than indulgence/enjoying the food”) and OrNe (six items; e.g., “My thoughts constantly revolve around healthy nutrition and I organize my day around it”). Both factors correlated to 0.65. Cronbach's alpha was 0.80 and 0.87 for DOS HeOr and DOS OrNe, respectively.

#### EHQ and DOS

The ESEM model that tested the latent structure of the combined EHQ and DOS scores showed an adequate fit (CFI = 0.959, TLI = 0.949, RMSEA = 0.060). The four factors replicated those found when questionnaires were analyzed one by one. Most importantly, the correlation between EHQ and DOS HeOr was 0.82; for EHQ and DOS OrNe, that correlation was 0.79.

### Association between orthorexia scales and lifestyle habits and sociodemographic variables

Table 4 presents the Pearson and partial correlations. The pattern of results is clear. HeOr showed a small negative relation with unhealthy consumptions (alcohol, smoking, and waterpipe), and the relationship was broader when controlling for OrNe (mean zero-order correlation = − 0.08, mean partial correlation = − 0.21). Oppositely, OrNe showed a small positive relation with unhealthy consumptions (alcohol, smoking, and waterpipe), and the relationship was broader when controlling for OrNe (mean zero-order correlation = 0.15; mean partial correlation = 0.24). No orthorexic dimensions showed an association with physical activity (maximum correlation = 0.02). When only considering alcohol, smoking, and waterpipe, the correlation sizes of the EHQ and DOS scores with HeOr were equivalent (mean partial correlation for EHQ = − 0.21; mean partial correlation for DOS = − 0.20), the associations were higher for EHQ scores with OrNe (mean partial correlation for EHQ = 0.29; mean partial correlation for DOS = 0.18).

**Table 4:** Zero order and partial correlations between variables

	AUDIT	FTND	LWDS	PA	Age	Gender
EHQ						
HeOr						
Zero order	.01	− **.15**	.02	.02	.05	**.15**
Partial	**−** **.18**	− **.30**	− **.15**	.02	**.20**	**.11**
OrNe						
Zero order	**.25**	**.14**	**.23**	.01	− .06	− .02
Partial	**.30**	**.29**	**.28**	.00	− **.14**	− **.11**
DOS						
HeOr						
Zero order	− **.11**	− **.11**	− **.12**	.02	.03	**.09**
Partial	− **.21**	− **.18**	− **.20**	.02	**.10**	.06
OrNe						
Zero order	**.11**	.06	.08	.02	− .05	.02
Partial	**.21**	**.15**	**.17**	.00	− .04	− .07

EHQ, Eating Habits Questionnaire; DOS, Dusseldorf Orthorexia Scale; AUDIT, Alcohol Use Disorders Identification Test; FTND, Fagerstrom Test for Nicotine Dependence; LWDS, Lebanon Waterpipe Dependence Scale; PA, Physical Activity. Gender was coded with a dummy variable where men = 0 and women = 1. For the associations with gender, the participant that did not report it was removed. Bold values correspond to statistically significant correlations (*p* < .05). Partial correspond to partial correlations controlling for the other orthorexia dimension

With respect to age, all the zero-order associations with orthorexia scores were not statistically significant (maximum |*r*|= 0.06). Regarding gender and considering HeOr, both women and men showed statistically significant differences with women presenting slightly higher mean scores (rs as small as 0.09 and 0.15). For OrNe, none of the comparisons between genders were statistically significant (rs equal to − 0.02 and 0.02).

## Discussion

The current study had two essential goals. The first goal of this study, needed to address the second one correctly, was to validate two questionnaires for the assessment of orthorexia in Arabic. Regarding the internal structure of both EHQ and DOS, evidence favored the bi-dimensionality of orthorexia. Both questionnaires presented two theoretically interpretable factors. The labels of Healthy Orthorexia (HeOr) and OrNe were borrowed from Barrada and Roncero [62]. While OrNe retains the interpretation of this disorder, key elements of HeOr are a “healthy interest in diet, healthy behavior with regard to diet, and eating healthily as part of one’s identity” [21]. Previous studies showed that OrNe is positively associated with psychopathology, whereas HeOr is independent and even inversely related to psychopathology [62, 63]. Also, motives predicting food choices for OrNe and HeOr are quite different [21].

In our study, some items were simultaneously tapping both dimensions of orthorexic tendencies, and shorter versions were proposed to offer instruments with a more explicit structure. The final version still presented high reliabilities.

Also, the two questionnaires presented an important convergent validity, as the dimensions with the same interpretation correlated around 0.80, a value in line with that of Meule et al. [33] (although there a single total score was computed for both EHQ and DOS). Although further research is needed to clarify the similarities and differences between OrNe and HeOr as measured by the EHQ and the DOS, both questionnaires presented a considerable overlap both in terms of theoretical interpretation and observed scores. The EHQ scores showed higher associations with unhealthy habits. It is not clear why such results were found.

It is noteworthy that almost every study analyzing the internal structure of the EHQ and the DOS scores has reached different conclusions. Several shortened versions of the EHQ (ours and the one from Godefroy et al. [32]) are currently available with a different number of factors (from two to four). For the DOS, our shortened version adds to solutions from unidimensional to three-dimensional structures. Our proposed shortened versions can be considered, tentatively, as valid and reliable. Valid as its structure shows concordance with a relevant theoretical framework in the area of orthorexia, that that distinghishes between OrNe and HeOr and showed adequate convergent validity. Reliable as the internal consistencies seemed adequate. However, further effort is needed to clarify how to assess orthorexia. The proposed structure and composition of the DOS and EHQ should be cross-validated with additional samples. Also, new instruments have been proposed, like the Teruel Orthorexia Scale [62] and Orthorexia Nervosa Inventory [64]. Importantly, the Teruel Orthorexia Scale assesses the two dimensions of orthorexia, and that structure has been replicated in different samples and countries [65–68]. Going further than which specific instrument should be used, a relevant point from our results is that, in any approach to measuring OrNe, HeOr has to be considered.

The second objective was to evaluate the correlation between OrNe (and now also HeOr) and lifestyle habits and sociodemographic variables. For this purpose, the convenience of adequately separating HeOr and OrNe becomes clear. Our results showed that while HeOr was negatively associated with the consumption of unhealthy substances (higher alcohol use disorder, cigarette, and waterpipe dependence), OrNe was positively correlated with these behaviors. After controlling for OrNe or HeOr (partial correlations), the associations were even higher; the pattern of associations was more explicit, showing the importance of considering the multidimensional structure of orthorexia. The studies conducted to date do not provide consistent data about the relationship between substance use, physical activity, and OrNe. Most of them do not find a significant relationship between substance use and the presence of OrNe, whereas results regarding physical activity are contradictory [69]. However, it is noteworthy that these studies were conducted with instruments of doubtful validity, such as the ORTO-15, without distinguishing between OrNe and HeOr. The ORTO has been criticized for its psychometric properties, low internal consistency and limited content validity [24, 70]. Therefore, the relationship with substance use (inverse with OrNe and direct with HeOr) found in our study could be masked in other research. When using the EHQ or the DOS, total scores not taking into account the bi-dimensionality of the instruments should be used with caution. The weak association between gender and age with orthorexia scores is in agreement with previous results [40, 63].

Overall, the negative association between OrNe and healthy lifestyle seems inconsistent with the same ideas behind OrNe. In theory, if the final goal of people with high OrNe tendencies is to maintain a healthy diet to achieve a healthy body, a generally healthy lifestyle should be expected (although convergent results have been recently presented) [68]. However, this incongruence can be explained by interpreting OrNe as a disorder analogous to eating disorders. OrNe could be understood from the classical view of Crisp for anorexia nervosa [71] or the transdiagnostic model for eating disorders proposed by Fairburn, Cooper, and Shafran [72, 73]. From this point of view, food is used as a tool to control the anxiety and achieve the sense of control to master insecurity and weakness, and lack of control in other aspects of life, trying to reach the “perfect diet” in OrNe, and the “perfect body” in the case of eating disorders, both culturally driven. In this sense, to reach the perfect diet as naturally as possible becomes a goal in itself, having nothing to do with achieving physical health. This idea is consistent with the association found in previous studies of OrNe with measures of psychopathology, such as negative emotionality [62, 63] or with emotional eating [63], thus explaining the presence of contradictory behaviors that are associated with emotional distress, such as tobacco and alcohol abuse, as often seen in patients with eating disorders [74, 75]. This hypothesis would be in line with studies relating OrNe to eating disorders symptomatology [1, 62, 76]. However, further studies are necessary to explore OrNe etiopathogenesis.

Moreover, HeOr would be related more directly to a general personal goal of a healthy life. While people with high levels of OrNe worried about eating healthy while exhibiting lower healthy behaviors, people with high levels of HeOr showed healthier life choices (except for physical activity).

### Limitations and strengths

This study presents several limitations. First, the present study is correlational without follow-up measurements. Its cross-sectional design did not allow to draw conclusions on causal relationships. Second, the recruitment method (people entering a pharmacy) may have introduced socioeconomic bias, so findings cannot be generalized to the whole population. Third, given that physical activity was measured based on self-reported data, it was not possible to compute any estimator of reliability, so the almost zero association with orthorexia due to measurement problems cannot be ruled out. Fourth, when developing short forms, it is advisable to test them with new, independent samples, which was not the case in our study. Fifth, the criteria used to remove participants due to missing values was arbitrary. We tried to discard this relevant problem by inspecting the results when using other criteria and found that the pattern of results was consistent. Finally, the influences of any cultural and linguistic differences on the results of this study were not checked.

Despite these limitations, our study has several strengths worth to be highlighted. It is among the few exceptions conducted with an Eastern population. Moreover, our sample was varied in terms of sociodemographic characteristics, unlike other studies conducted only among university students. Finally, this study could provide an in-depth psychometric analysis of two orthorexia questionnaires.

## Conclusions

For a long time, OrNe and HeOr have been considered as essentially equivalent terms. Our results emphasize the idea that further attention should be paid to the multidimensional structure of orthorexia, as OrNe and HeOr present an opposite pattern of associations with healthy behaviors. An OrNe etiopathogenesis common to eating disorders can explain these differences. Future studies should analyze the etiology of OrNe to understand this disorder better while carefully considering what each orthorexia questionnaire measures.

## Data Availability

The datasets generated and/or analyzed during the current study are not publicly available as per their institutions policies but are available from the corresponding author on reasonable request.

## Abbreviations

EHQ: Eating Habits Questionnaire
DOS: Dusseldorf Orthorexia Scale
OrNe: Orthorexia nervosa
HeOr: Healthy Orthorexia
AUDIT: Alcohol Use Disorders Identification Test
FTND: Fagerström Test for Nicotine Dependence
LWDS: Lebanon Waterpipe Dependence Scale
ESEM: Exploratory structural equation models
